# Polyethylene Oxide-Based Composites as Solid-State Polymer Electrolytes for Lithium Metal Batteries: A Mini Review

**DOI:** 10.3389/fchem.2020.00640

**Published:** 2020-08-11

**Authors:** Shuangshuang Zhao, Qinxia Wu, Wenqing Ma, Lishan Yang

**Affiliations:** ^1^Key Laboratory of Chemical Biology & Traditional Chinese Medicine Research (Ministry of Education of China), National and Local Joint Engineering Laboratory for New Petrochemical Materials and Fine Utilization of Resources, Key Laboratory of the Assembly and Application of Organic Functional Molecules of Hunan Province, Hunan Normal University, Changsha, China; ^2^School of Materials Science and Engineering, Qilu University of Technology (Shandong Academy of Sciences), Jinan, China

**Keywords:** solid-state polymer electrolytes, polyethylene oxide, lithium metal batteries, ionic conductivity, electrochemical stability

## Abstract

Solid-state polymer electrolytes (SPEs) have great processing flexibility and electrode–electrolyte contact and have been employed as the promising electrolytes for lithium metal batteries. Among them, poly(ethylene oxide) (PEO)-based SPEs have attracted widespread attention because of easy synthesis, low mass density, good mechanical stability, low binding energy with lithium salts, and excellent mobility of charge carriers. In order to overcome the low room-temperature ionic conductivity and the poor thermodynamic stability in high-voltage devices (>4.2 V) of the PEO materials, composition modulations by incorporating PEO with inorganic and/or organic components have been designed, which could effectively enable the applications of PEO-based SPEs with widened electro-stable voltage ranges. In this mini review, we describe recent progresses of several kinds of PEO composite structures for SPEs, and we compare the synthesis strategies and properties of these SPEs in lithium batteries. Further developments and improvements of the PEO-based materials for building better rechargeable batteries are also discussed.

## Introduction

Lithium ion batteries (LIBs) are critical for large-scale applications, including power sources for 3C electronics, portable devices, and electric vehicle (Liu et al., 2019b). The innovation of advanced batteries with higher energy/power densities, longer cycling life, and, especially, guaranteed levels of safety at a satisfactory cost is crucially needed (Manthiram et al., 2017; Liu et al., 2018). As the most potential anode material, Li-metal possesses advantages such as high theoretical capacity (3,860 mA h g^−1^), negative potential [−3.04 V vs. standard hydrogen electrode (SHE)], and low density (~0.59 g cm^−3^) (He et al., 2018; Zhao C. Z. et al., 2019). However, Li-metal batteries (LMBs) with liquid electrolytes cannot coordinate a high energy density with excellent electro-stability for real applications (Choudhury et al., 2019b). The robustness and integrity of the electrode–electrolyte interface through cycling ensure the efficacy of the whole cell (Maleki Kheimeh Sari and Li, 2019). To address these issues, solid-state polymer electrolytes (SPEs) in the replacement electrolytes have already demonstrated feasibility and superiority in lithium secondary batteries (e.g., solid-state LMBs, lithium-sulfur batteries, and lithium-gas batteries) (Xiao et al., 2019; Zhou et al., 2019; Zhao et al., 2020).

SPEs are generally composed of polymeric solid host and lithium salt without the use of liquid solvents. The construct of SPEs was derived from the discovery by Fenton et al. (1973) that alkali metal salts dissolved in polyethylene oxide (PEO) could form conductive complexes (Fenton et al., 1973). In general, various lithium-ion conductive polymer materials, such as PEO (Zhao Q. et al., 2019), poly(acrylonitrile) (PAN) (Wang et al., 1996), poly(methyl methacrylate) (PMMA) (Appetecchi et al., 1995), and poly(vinylidene fluoride) (PVDF) (Choe et al., 1995), have been employed as SPEs for the development of solid-state LMB batteries. Generally, a preferred SPE, used to replace the electrolyte in lithium batteries, should contain a high dielectric host polymer and a lithium salt with a low lattice energy. In this regard, the polymers with polar functional groups are required for consideration from the dissociation and transportation of the lithium ions (Liang et al., 2019). Among all polymers, PEO is widely studied because of its excellent salt-solvating ability and electrode interfacial compatibility. From the above considerations, we selected and reviewed PEO-based SPEs (Xu et al., 2019; Nie et al., 2020; Qiu et al., 2020).

However, the room-temperature (RT) ionic conductivity of PEO-based SPEs is lower than that of the liquid-based counterpart. Moreover, the batteries constructed with PEO-based SPEs usually suffer from Li dendrite because of the poor mechanical strength of the polymer matrix. Therefore, future studies of PEO-based SPEs should focus on the improvement of the mechanical properties for preventing Li dendrite formation and increasing electrochemical stability of PEO-based SPEs. By compositing with inorganic solid oxides/organic molecules, as-obtained composite PEO-based polymer electrolytes show a high ionic conductivity and are stable under relative high voltage (Liang et al., 2019). For inorganic-PEO SPEs, the hybrid electrolyte consists of a soft polymer PEO electrolyte and a rigid inorganic solid-state electrolyte (SSE), which has a relatively high ionic conductivity at RT (Choudhury et al., 2019a; Qiu et al., 2020). As for organic material-modified PEO SPEs, chemical strategies (copolymerization and crosslinking) frustrate crystallization to enable acceptable ionic conductivities at temperatures near ambient or within the thermal window of applications, including in transport applications (Liu et al., 2019a; Zhao et al., 2020). Moreover, complex synergistic optimization is the primary efficient method of improving the antioxidant stability of systems with PEO-based electrolytes because of its high mechanical strength and high ionic conductivity (Feng et al., 2018; Zhang et al., 2019).

The amorphous PEO domain benefits Li^+^ diffusion and is preferred as compared with the crystalline one. With the addition of filler particles, the PEO SPEs show increased mechanical strength and thermal stability and can thus suppress crystallization and facilitate the dissociation of the salt with an improved ionic conductivity (Zhao et al., 2020). With PEO-based electrolyte, the all-solid-state Li|LiFePO_4_ (LFP) cell, Li|LiCoO_2_ cell, and Li|LiNi_1−x−*y*_Mn_*x*_Co_*y*_O_2_ (NMC) cell all show excellent cycling stability and rate capability. The PEO-based SPEs are of great significance to improve the practical application of hot ternary cathode materials (Wang C. et al., 2019; Wang X. et al., 2019; Xu et al., 2019).

In this work, we review the recent progress in the synthesis strategies and polymer engineering of PEO-based electrolytes. We specifically compare the ion conductivity and electro-stability of different compound electrolytes and thus provide a certain reference for structural optimization of the PEO-based SPEs.

## PEO Polymer Solid Electrolytes Based on Different Compositions

PEO-based composite SPEs show a high probability in all-solid-state lithium batteries, including inorganic-PEO composite, organic-PEO composite, and other complex composite electrolytes. The relevant synthesis/modification avenues and their performance/characteristics are summarized in the following sections.

### Inorganic-PEO Composite SPEs

In initial studies, PEO electrolyte is employed in solid-state LMBs but shows a low conductivity and inferior thermal stability. It is found that the increment of lithium salts within the polymeric matrix can lead to an improved ionic conductivity, accompanied by a degradation of mechanical strength and electro-stability of the PEO-based SPEs (Lopez et al., 2019). Integration of nanostructured inorganic fillers (including metal oxide, neotype lithium salts, and some oxide solid-solutions) into polymeric solid host, named as “inorganic-PEO composite,” has emerged as an effective approach to design SPEs with the most needed improvements.

**Figure 1:**
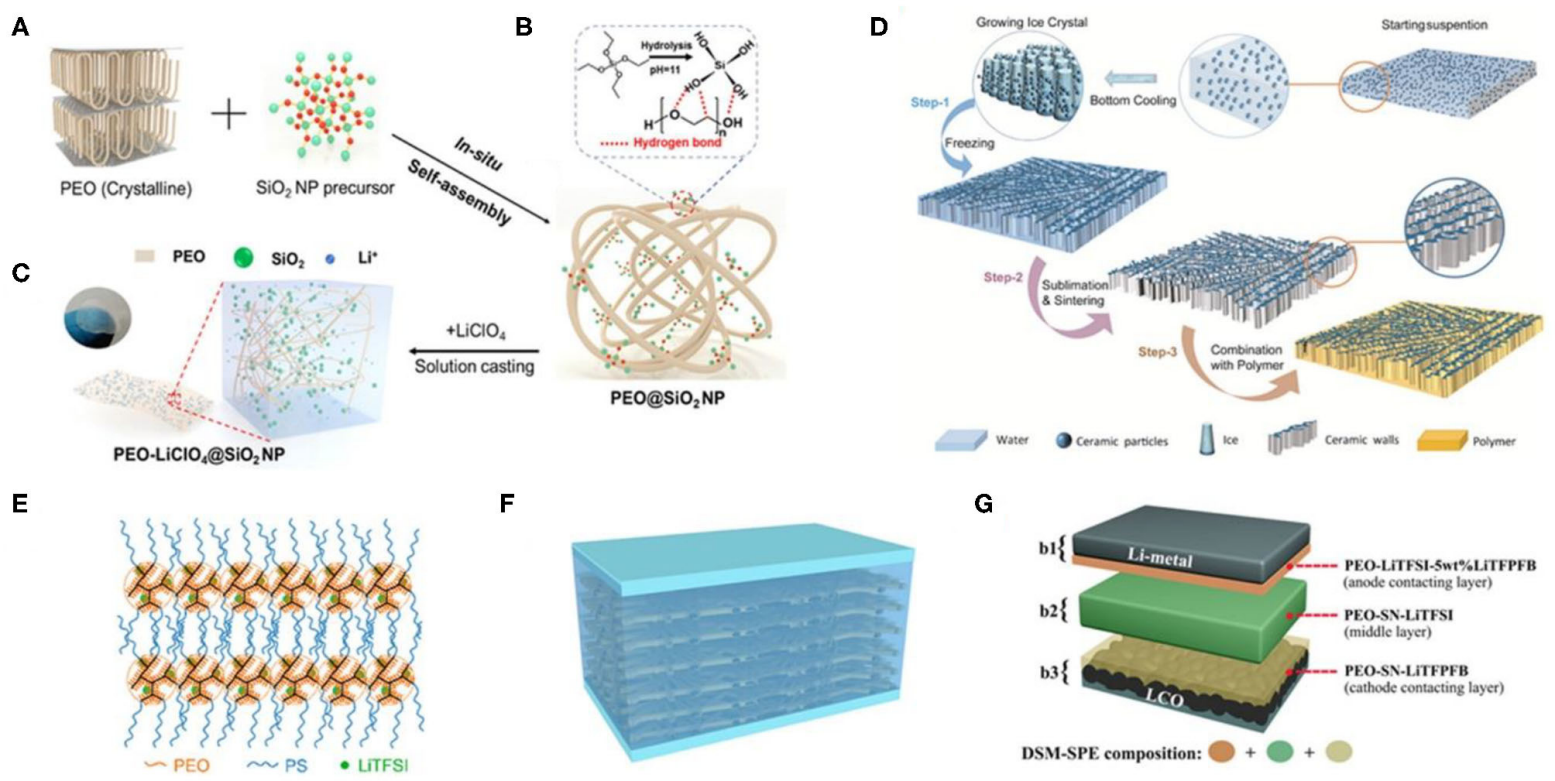
**(A–C)** Synthetic routes of the SiO_2_-PEO–LiClO_4_ solid-state polymer electrolytes (SPEs). **(D)** The schematic preparation process of the ice-templated Li_1.5_Al_0.5_Ge_1.5_(PO_4_)_3_ (LAGP)-PEO SPEs. Reproduced with permission from Wang X. et al. (2019) and Xu et al. (2020). **(E)** Schematic structure of the lamellar PS–PEO–LiTFSI SPEs. Reproduced with permission from Chen et al. (2019). **(F)** Schematic of the PEO|PEO–perovskite|PEO composite solid electrolyte. **(G)** Schematic of the multilayered solid polymer electrolyte (DSM-SPE). The bilateral layers are *in situ* formed on the surfaces of electrodes. Reproduced with permission from Wang C. et al. (2019) and Liu K. et al. (2019).

Basically, composite casting strategies can be divided into two-step solution casting and *in situ* casting combined with fillers. To date, the *in situ* approach, which possesses good distribution of nanofillers and is scalable for practical application, is commonly used for the syntheses of composite SPEs (Yap et al., 2013; Xu et al., 2020). Two-step reactions usually include the formation of the fillers (step I) and the incorporation operation (step II). For example, in a two-step synthesis process, self-synthesized MgAl_2_O_4_ nanoparticles were incorporated into PEO polymers through a rapid hot press procedure (Angulakshmi et al., 2013). For example, Al_2_O_3_-PEO SPEs synthesized via the *in situ* method could get a conductivity of 2.970 × 10^−5^ S cm^−1^ with microsized Al_2_O_3_ fillers at RT; however, composite SPEs with Al_2_O_3_ (<50 nm) displayed rougher surface structures and a reduced conductivity of 4.843 × 10^−6^ S cm^−1^ (Yap et al., 2013). Generally, the conductivity of the Al_2_O_3_-PEO SPEs can be improved with a small Al_2_O_3_ filler particle, which can probably have a more powerful impact on the immobilization of the long polymer chains. However, the fine (nanosized) filler grains would be too close to each other, inducing the blocking effect of the filler grains with enhanced immobilization and leading to the decrease in the ionic conductivity. Recently, through a rigid–flexible coupling technology, nano-SiO_2_ particles were incorporated into 3D PEO networks to *in situ* construct SiO_2_-PEO SPEs (shown in Figures 1A–C). SiO_2_-PEO SPEs show an outstanding RT ionic conductivity (σ ≈ 1.1 × 10^−4^ S cm^−1^) along with dramatically improved solid–solid interface stabilization and excellent high-temperature capability (~90 mA h g^−1^ after 100 cycles under 2 C at 90°C) (Xu et al., 2020).

In recent years, neotype lithium salts (inorganic ion conductors) are regarded as the most promising filler materials for inorganic-PEO SPEs, which can effectively enhance the battery properties in the electrochemical aspect. Phosphate ion conductor nanomaterials such as Li_1.5_Al_0.5_Ge_1.5_(PO_4_)_3_ (LAGP) (Wang X. et al., 2019) and Li_1+x_Al_*x*_Ti_2−x_(PO_4_)_3_ (LATP) (Zhai et al., 2017) are reported to present optimal ionic conductivities and superior flexibilities than do the SPEs before compounding. The inorganic particles were first dispersed into water and cast onto a substrate. Thereafter, the bottom of the dispersion is slowly cooled down and forms a vertical temperature gradient with ice nucleated from the bottom of the suspension, during which the ceramic particles are compelled to form vertically aligned structures. After ice sublimation, inorganic particles are sintered together to form vertically aligned walls (Wang X. et al., 2019). For example, PEO polymer combined with vertically aligned inorganic walls (shown in Figure 1D) shows an RT ionic conductivity level of 10^−4^ S cm^−1^, which is three to six times higher than that of the PEO electrolyte with ceramic nanoparticles randomly dispersed inside. For the LLTO-PEO SPEs, the prepercolating structure in Li_0.35_La_0.55_TiO_3_ (LLTO) could accelerate Li-ion conduction (σ = 0.88 × 10^−4^ S cm^−1^) via its successive ion pathways, which could also further avoid agglomeration of particles (Bae et al., 2018). As a Li-ion fast ionic conductor, LLZO also owns remarkable electrochemical stability (even under 6.0 V). Thus, the Li symmetric cells using LLZO-PEO SPEs can stably cycle for 1,000 h without short-circuiting even at 60°C, which is due to the highly packed LLZO nanowires inside the SPEs and the uniform deposition of lithium metal (Wan et al., 2019). Furthermore, other lithium salts, such as sulfide (Li_3_PS_4_) and borate [lithium bis(modified imidazole)borate] (LiBMB), have been successfully used to ameliorate flexible PEO-based SPEs. Li_3_PS_4_ particles exhibit uniformly nanosized morphologies, and the particle diameter of the Li_3_PS_4_ is about 400–700 nm. *In situ* synthesis of Li_3_PS_4_ nanoparticles within the PEO matrix possesses good distribution of nanofillers. β-Li_3_PS_4_ glass-ceramic is a Li superionic conductor whose conductivity is higher than 10^−4^ S cm^−1^ at RT with a relatively stable electrochemical property (Chen et al., 2018). LiBMB-PEO SPEs display a highly ordered ionic pathways [the quasi-period value (*T*_p_) is lower than 100 ns] associated with a high electro-stable potential of up to 7.2 V at 60°C (Yuan et al., 2019). The LiFePO_4_|LiBMB-PEO SPEs|Li cell delivers an initial discharge capacity of 145.5 mA h g^−1^ at 0.1 C and ultra-high capacity retention (98.5% after 60 cycles), revealing good reliability of the produced cells.

Besides the aforementioned lithium salt, several metal oxide solid solutions such as MgAl_2_O_4_ have been supplied in the PEO-based composite SPEs (Angulakshmi et al., 2013). In this two-step synthesis case, the self-synthesized MgAl_2_O_4_ nanoparticles were incorporated with PEO polymer through a rapid hot press procedure. The assembled all-solid-state cells deliver a discharge capacity approaching 110 mA h g^−1^ after prolonged cycling (up to 100 cycles, 70°C), which renders them to be qualified for practical battery applications.

In short, inorganic-PEO SPEs show considerable superiority with an improved ionic conductivity, enhanced mechanical strength, and obvious accessibility for enlarging the preparation. Along with the introduction of inorganic fillers, the possible conductive mechanisms in the composite SPEs, especially with neotype lithium salts as the fillers, need more comprehensive and in-depth research. Also, improving the RT ionic conductivities of the current inorganic-PEO SPEs to the liquid-electrolyte level (10^−3^-10^−2^ S cm^−1^) becomes the most urgent work.

### Organic-PEO Composite SPEs

Compared with inorganic-PEO composite SPEs, organic-PEO composite SPEs have been widely studied in previous works (Lopez et al., 2019). Related organic fillers can be briefly classified as metal-organic frameworks (MOFs), simple organisms, and molecular polymers.

MOF materials have been investigated for extensive application in the fields of electrochemical catalysis, electrodes, and electrolyte because of their high specific surface area and ordered microporous structure. For example, Zn_4_O(1,4-benzenedicarboxylate)_3_ (MOF-5) nanoparticles with a size of 20–30 nm were first synthesized and then incorporated into the polymer metric to form the MOF-PEO SPEs (Yuan et al., 2013). MOF-5 fillers show a strong absorbing ability for the solvent impurities and help SPEs to prevent those impurities from accumulating at the Li/SPEs interface. The reversible capacities of cells increase after filling MOF-5 particles as a result of reduced cell polarization of the electrode/SPE interface at both 60 and 80°C.

Also, simple organic fillers such as silicane (Mehdi et al., 2017; Mohanta et al., 2017) and carbonate (He et al., 2017) have been successfully introduced into the PEO-based SPEs, improving the ion conductivity and ameliorating the polymer crystallinity. For example, the monosilylated PEO precursor with the loose networks supplies higher segmental motion and amorphous networks, which are beneficial for a significantly enhanced capacity and faster Li-ion transmission (Mohanta et al., 2017). By a facile transesterification reaction, the carbonate-linked PEO SPEs could achieve a high yield with intrinsic amorphous nature and low glass transition temperature, which would be beneficial for getting a high ionic conductivity. Cycling performance of the cell with carbonate-linked PEO SPEs displays extremely stable capacity as well as coulombic efficiencies close to 100% up to 100 cycles at both 25 and 55°C.

Moreover, the ionic conductivity of a polymer electrolyte depends on the chain mobility. Various kinds of polymer fillers, such as polystyrene (PSt) (Niitani et al., 2005), PVDF (Deng et al., 2015), polyvinyl alcohol (PVA) (Jinisha et al., 2018), thermoplastic polyurethane (TPU) (Tao et al., 2017), and polystyrene (PS) (Chen et al., 2019), are discussed in the following portion. Copolymer PSt-PEO with microphase separation structure displays high tensile strengths (>3 MPa), which is high enough utilization in battery applications. PSt-PEO SPEs exhibit exceptionally high Li^+^ conductivity (3.03 × 10^−3^ S cm^−1^ at RT) and remarkable electrochemical stability (stable above 5.0 V vs. Li/Li^+^). A similar RT ionic conductivity has been reported in the study of PVA-PEO composite SPEs. The superhigh conductivity value is evaluated in the PSt-PEO and PVA-PEO SPEs, which is quite close to that of the liquid electrolytes.

Thermogravimetric analysis is constantly used to directly investigate the thermal stability of the polymer electrolytes. In the case of TPU-PEO SPEs (Tao et al., 2017), copolymer electrolyte shows a high conductivity (5.3 × 10^−4^ S cm^−1^ at 60°C) and notably enhanced thermostability even under 200°C. A similar conductivity and electrochemical improvements could be observed for PS-PEO SPEs (Chen et al., 2019). PS-PEO SPEs have a high storage modulus of 1.4 MPa at 60°C. The Li|PS-PEO SPE|Li cell can be cycled for 400 h at 0.1 mA cm^−2^ without any Li dendrite failure. The charge passed is 144 C cm^−2^ with an average voltage increase of 0.6 mV C^−1^ cm^2^. These results indicate that the Li/SPE interface is stable and that the mechanical strength of the SPEs is capable of suppressing Li dendrite growth. As illustrated in Figure 1E, PS arms would rearrange and entangle after phase separation, and then the lamellar structure would be reformed by alternating self-assembled PS and PEO layers. So far, several types of organic fillers of the PEO-based SPEs have been discussed in this portion, and more organic fillers will be explored and applied for high-performance flexibility SPEs.

### Other Complex Composite SPEs

Except for simplex compound fillers in PEO-based composite SPEs, multiphase composites, such as PEO-inorganic-organic, PEO-organic-organic, and other complicated ones, have also been widely explored to satisfy the need of practical application. These multiblock copolymers with various fillers show improved properties in different aspects.

PEO-inorganic-organic SPEs composite possess the combined advantages of PEO, inorganic and organic components. For instance, the PEO–Al_2_O_3_-Pr_4_NI (tetrapropylammonium iodide) composite SPEs show a high conductivity of 4.2 × 10^−4^ S cm^−1^ and an admirable PEO spherulitic crystallinity at 24°C with 5% Al_2_O_3_ filler (Bandara et al., 2017). The ceramic material Li_6.4_La_3_Zr_1.4_Ta_0.6_O_12_ (LLZT) was introduced to the PEO–succinonitrile (SN) system (Zha et al., 2018). The one containing 60 wt% of LLZT and 10 wt% of SN shows a high ion conductivity of 1.22 × 10^−4^ S cm^−1^ at 30°C and a wide electrochemical window of 5.5 V vs. Li/Li^+^. The SPEs composed of aluminate complexes (LiAl)–polyethylene glycol (PEG) and PEO could also be prepared via the common solution casting method (Feng et al., 2018). With PEO additive, the segmental mobility of the ether-chain bonded with Al atoms would be improved, providing PEO–LiAl–PEG hybrid SPEs with extra ionic pathways and high ionic conductivity. The new hopping transport mechanism was verified for the single Li-ion conductor system at the nanoscale.

PEO-organic-organic multiblock copolymer SPEs have recently garnered increasing attention because of their hard-separated segments and improved mechanical properties with their multiple microphase separated domains. A multiblock copolymer electrolyte composed of poly(butylene terephthalate) (PBT) and PEO alternating multiblock copolymers (mBCPs) (PBT-*b*-PEO-*b*-PBT)_*n*_, is synthesized by the cascade polycondensation-coupling ring-opening polymerization (PROP) method (Huang et al., 2017). The mBCP electrolyte shows a high ionic conductivity of 8.2 × 10^−4^ S cm^−1^ at 90°C and considerable mechanical properties. A gel polymer electrolyte composed of PEO, PMMA, and P(VDF-HFP) (PEMVH) is synthesized by adding some inorganic oxide fillers (Shi et al., 2018). The PEMVH-oxide filler electrolyte possesses porous and amorphous structures, in which the oxide fillers can promote the formation of the pores of the polymer matrix and the segmental motion of the polymer chain. This unique structure of the polymer membranes contributes to the high ionic conductivity and interfacial stability of hybrid SPEs.

In addition, multilayered composite PEO-based SPEs are also developed to give excellent mechanical stability, ionic conductivity, and interfacial compatibility for the all-solid-state lithium batteries. The flexible composite SPEs composed of PEO layers on both sides of the PEO–perovskite composite are actually an integrated sandwich structure, which is denoted as PEO|PEO-perovskite|PEO (shown in Figure 1F). The synthesis routes PEO and LiTFSI were dissolved into acetonitrile. The solution was then dropped onto the surface of the Li_0.33_La_0.557_TiO_3_ (LLTO) nanofiber mat. The wetted LLTO mat was predried in air for 6 h before drying in vacuum for 24 h at 65°C. Then, the membrane was turned to the other side, followed by repeating the wetting and drying processes to obtain the PEO–perovskite SPEs with an integrated sandwich structure. The 3D perovskite nanofiber network can enhance the ionic conductivity by offering Li-ion with abundant channels and improve the mechanical strength of the membrane (Liu K. et al., 2019). Besides, multilayered SPEs with a differentiated salt-based multilayered solid polymer electrolyte were synthesized via a facile slurry casting-drying method, which helped the Li symmetric battery to achieve a high capacity retention of 79.0% (after 300 cycles at 60 and 2°C) (Wang C. et al., 2019). The schematic of multilayered SPEs are displayed in Figure 1G. Every layer is purposefully designed to make full use of their respective advantages (i.e., the middle layer can provide a good ionic conductivity for the electrolyte), while the outer layers can enhance the interfacial contact and promote the formation of steady solid electrolyte interface (SEI) and cathode electrolyte interface (CEI) films. As compared with conventional PEO-based SPEs, the multilayer strategy is powerful to achieve high performance and solve electrolyte/electrode interfacial problems of the solid-state batteries.

**Figure 2:**
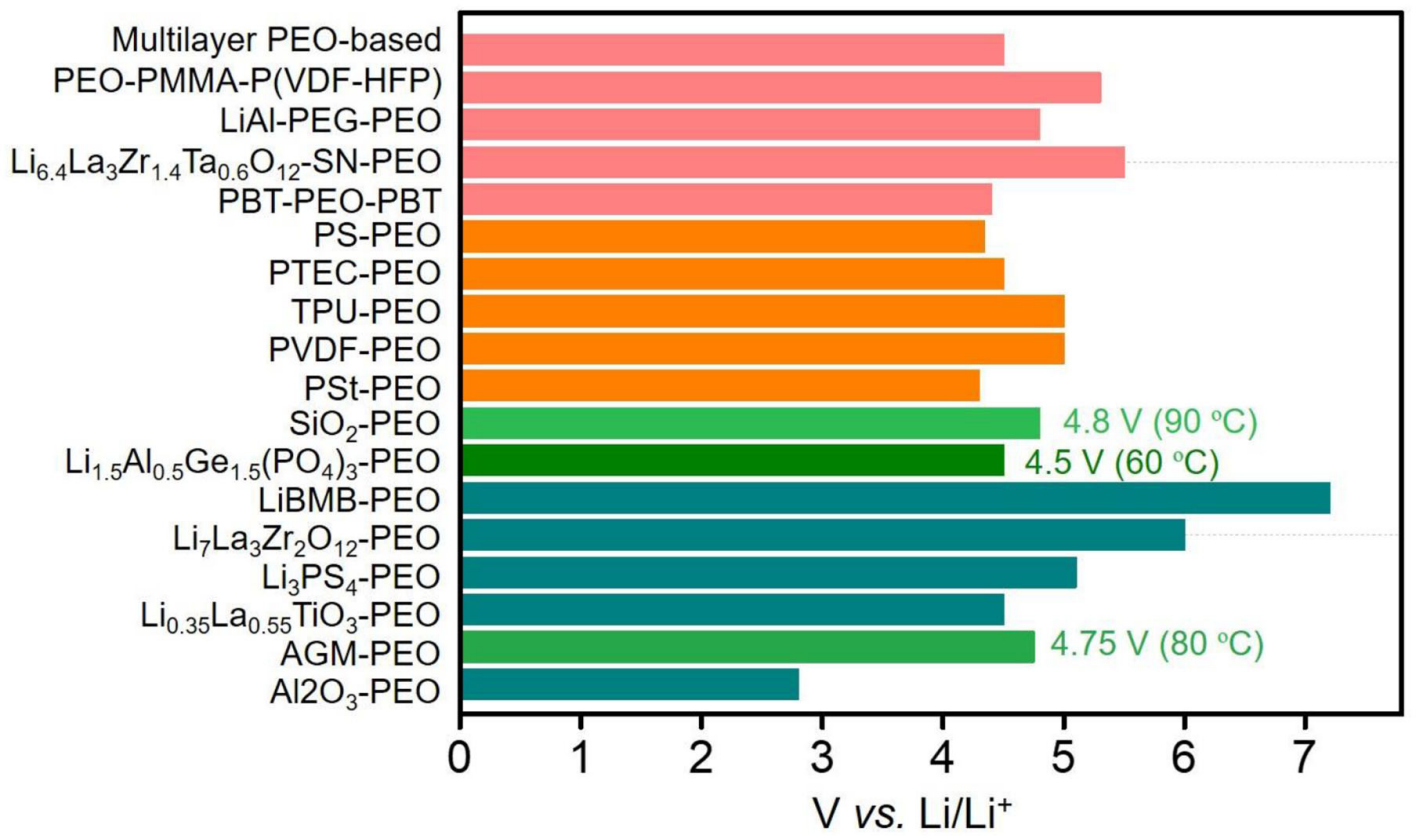
Calculated electrochemical stability windows for the polyethylene oxide (PEO)-based solid-state polymer electrolyte (SPE) candidates in a lithium metal battery. The inorganic-PEO hybrid SPEs are marked by green and dark cyan bars; the organic-PEO hybrid SPEs are marked with an orange bar; and the complex PEO-based hybrid SPEs are marked with a rose bar.

A summary of the electrochemical window and the ionic conductivity of PEO-based SPEs is presented in Figure 2 and Table S1. Most of them exhibit high electrochemical stable voltages (≥4.5 V) and ionic conductivity (≥1 × 10^−4^ S cm^−1^). Generally, PEO-organic and multiple complex SPEs show superior high-temperature conductivity and mechanical property. In general, the multiple complex electrolyte with high ionic transport and electrode–electrolyte interfacial stability is expected for future commercial application of solid-state LMBs.

## Conclusions

PEO-based SPEs have gained wide attention as promising electrolytes for lithium metal batteries (LMBs) because of their superior processing flexibility and electrode–electrolyte contact. Considerable research efforts have been devoted to improving the SPEs' limited room-temperature ionic conductivity and electrochemical stability of PEO. Inorganic and organic components have been introduced to the PEO-based SPEs to widen its electrochemical window of PEO. The inorganic fillers possess the advantages of low cost and high safety, while the organic ones can improve the SPEs' flexibility and change the polymer crystallinity with a crosslink or copolymerization approach. Moreover, by using multiple complex PEO-based SPEs, the LMBs can achieve much enhanced mechanical strength and electrode-electrolyte interfacial stability. At present, developing suitable PEO-based composite SPEs remains a challenge for the practical applications of high-performance LMBs with high safety and electro-stability.

## Author Contributions

LY supervised the implementation of the project. LY and SZ conceived the idea. SZ, QW, WM, and LY analyzed the data and wrote the manuscript. All authors contributed to the article and approved the submitted version.

## Conflict of Interest

The authors declare that the research was conducted in the absence of any commercial or financial relationships that could be construed as a potential conflict of interest.
