# Supplementary material for: Polyethylene Oxide-Based Composites as Solid-State Polymer Electrolytes for Lithium Metal Batteries: A Mini Review
Source: Front Chem. 2020 Aug 11;8:640. doi: 10.3389/fchem.2020.00640 (PMC7431671; doi:10.3389/fchem.2020.00640)
Supplement: Supplementary file 1 [file Table_1.DOCX]

Supplementary Material

**PEO-based composites as solid-state polymer electrolytes for lithium metal batteries: a mini review**

Shuangshuang Zhao ^a^, Qinxia Wu ^a^, Wenqing Ma ^b^, Lishan Yang ^a,^*

*^a^ Key Laboratory of Chemical Biology & Traditional Chinese Medicine Research (Ministry of Education of China), National and Local Joint Engineering Laboratory for New Petrochemical Materials and Fine Utilization of Resources, Key Laboratory of the Assembly and Application of Organic Functional Molecules of Hunan Province, Hunan Normal University, Changsha, Hunan 410081, P.R. China*

*^b^ School of Materials Science and Engineering, Qilu University of Technology (Shandong Academy of Sciences), Jinan 250353, P. R. China.*

Corresponding author:

[lsyang.chemistry@gmail.com](mailto:lsyang.chemistry@gmail.com) (L.S.Y.)

Tel./Fax.: +86 731 88872531

**Table S1** Ionic conductivities of PEO-based composite polymer electrolytes with various fillers.

| Fillers | Lithium salts | Ionic conductivity  (S cm^‒1^) | Temperature (ºC) | Ref. |
| --- | --- | --- | --- | --- |
| Al_2_O_3_ | LiCF_3_SO_3_ | 4.8×10^‒6^ | 25 | (27) |
| SiO_2_ | LiClO_4_ | 1.1×10^‒4^ | 30 | (28) |
| Li_1.5_Al_0.5_Ge_1.5_(PO_4_)_3_ | LiTFSI | 1.67×10^‒4^ | room temperature | (24) |
| Li_1+x_Al_x_Ti_2−x_(PO_4_)_3_ | LiClO_4_ | 0.52×10^‒4^ | room temperature | (30) |
| Li_0.35_La_0.55_TiO_3_ | LiTFSI | 0.88×10^‒4^ | room temperature | (31) |
| Li_7_La_3_Zr_2_O_12_ | LiTFSI | 2.39×10^‒4^ | 25 | (32) |
| Li_3_PS_4_ | Li_3_PS_4_ | 8.01×10^‒4^ | 60 | (33) |
| LiBMB | LiBMB | 0.45×10^‒3^ | 30 | (34) |
| Silsesquioxane | LiTFSI | 4.4×10^‒4^ | 80 | (36) |
| PSH | LiCF_3_SO_3_ | 7.2×10^‒5^ | room temperature | (37) |
| PDEC | LiTFSI | 1.12×10^‒5^ | 25 | (38) |
| PSt | LiClO_4_ | 2×10^‒4^ | 30 | (39) |
| PVDF | LiClO_4_ | 3.03×10^‒3^ | room temperature | (40) |
| PVA | LiClO_4_ | 3.5×10^‒3^ | room temperature | (41) |
| TPU | LiTFSI | 5.3×10^‒4^ | 60 | (42) |
| PS | LiTFSI | 4×10^‒4^ | 60 | (43) |
| Al_2_O_3_+Pr_4_N^+^I^‒^ |  | 4.2×10^‒4^ | 24 | (44) |
| Li_6.4_La_3_Zr_1.4_Ta_0.6_O_12_+SN | LiTFSI | 1.22×10^‒4^ | 30 | (45) |
| LiAl-PEG | LiClO_4_ | 2.6×10^‒4^ | 100 | (23) |
| PBT-PEO-PET | LiTFSI | 8.2×10^‒4^ | 90 | (46) |
| PMMA+P(VDF-HFP) | LiPF_6_ | 8.1×10^‒5^ | 25 | (47) |
| PEO-Perovskite-PEO | LiTFSI | 1.6×10^‒5^ | 24 | (48) |
| Multilayered PEO | LiTFSI | 5×10^‒4^ | 30 | (25) |
